# Association between Dietary Zinc Intake, Serum Zinc Level and Multiple Comorbidities in Older Adults

**DOI:** 10.3390/nu15020322

**Published:** 2023-01-09

**Authors:** Sirasa Ruangritchankul, Chutima Sumananusorn, Jintana Sirivarasai, Wutarak Monsuwan, Piyamitr Sritara

**Affiliations:** 1 Division of Geriatric Medicine, Department of Medicine, Faculty of Medicine, Ramathibodi Hospital, Mahidol University, Bangkok 10400, Thailand; 2 Master of Science Program in Nutrition, Faculty of Medicine, Ramathibodi Hospital and Institute of Nutrition, Mahidol University, Bangkok 10400, Thailand; 3 Graduate Program in Nutrition, Faculty of Medicine, Ramathibodi Hospital, Mahidol University, Bangkok 10400, Thailand; 4 Department of Medicine, Faculty of Medicine, Ramathibodi Hospital, Mahidol University, Bangkok 10400, Thailand

**Keywords:** older adults, serum zinc concentration, dietary zinc intake, multimorbidity

## Abstract

Zinc is one of the essential micronutrients in the geriatric population, but the importance of zinc status and dietary zinc intake has been poorly characterized. We aimed to explore the relationships among dietary zinc intake, serum zinc concentrations and multimorbidity in a cross-sectional study of 300 employees of Electric Generating Authority of Thailand aged ≥ 60 years. Comprehensive questionnaires were completed, and clinical and laboratory assessments were performed. Factors associated with low serum zinc concentrations were identified using multivariate multinomial logistic regression analyses. The mean serum zinc level was 80.5 (12.8) µg/dL. After adjustment for baseline characteristics, being female and having been in education for ≤12 years were independent risk factors for the lowest tertile (T1) of serum zinc. After additional adjustment for clinical and biochemical parameters, there was a significant association between depression (Thai Geriatric Depression Scale-15 score > 5) and low serum zinc levels (T1 vs. T3, odds ratio (OR): 2.24; 95% confidence interval (CI): 1.06–4.77). Furthermore, as serum albumin increased, serum zinc concentration substantially increased (T1 vs. T3, OR: 0.01; 95% CI: 0.002–0.070). Therefore, the early detection of risk factors and the further management of depression and low serum albumin may assist physicians in preventing low serum concentrations.

## 1. Introduction

In the last decade, several countries, including Thailand, have become aging societies owing to the rapid increase in the number of older persons with longer life expectancy [1]. Globally, the number of people of ≥60 years in 2020 was almost 1 billion, and this is predicted to more than double and reach 2.1 billion by 2050 [1]. In Thailand, the number of older people aged ≥ 60 years was approximately 12 million in 2020 and is expected to reach 20 million by 2040 [2]. Aging individuals are more liable to experience multiple chronic diseases and geriatric conditions. The most common comorbidities in older adults, such as diabetes mellitus, hypertension, dyslipidemia and ischemic stroke, are associated with serum zinc concentration. According to recent literature reviews, the pathophysiology of these chronic diseases is associated with increased oxidative stress and free radicals. Zinc is an essential intracellular trace element and acts as a potent antioxidant [3,4,5,6]. Besides, inflammatory process is also related to serum zinc level in the development of age-related chronic diseases [7]. Furthermore, physical activity should be taken into account in the geriatric population [8,9]. It has been found that impaired physical activity may be correlated with serum zinc levels [10,11,12]. Zinc deficiency in the geriatric population may be caused by age-related physiological changes, including reduced saliva secretion, decreased smell and taste perception [13], degenerative changes in the intestinal microvilli [14] and deterioration of cognitive or muscle function [15]. These physiological alterations contribute to inadequate zinc intake and zinc malabsorption, leading to zinc deficiency. Worldwide, the prevalence of zinc deficiency ranges from 20% to 40% of the total population, whereas one third of older adults are at greater risk of zinc deficiency [16,17].

Zinc is an essential micronutrient, because it plays a major role in metabolic function and cellular structure [18,19]. Zinc must be obtained externally through diet and is absorbed by the intestine. The richest food sources of zinc include beef, pork, fish, nuts, seeds and whole-grain cereals [20]. Therefore, inadequate intake of food rich in zinc causes a deficiency. According to a recent study, approximately 25% of the global population was vulnerable to inadequate zinc intake [21]. Zinc deficiency has adverse effects on health outcomes through impairment of the immune response, psycho-cognitive problems and delayed wound healing [15,21,22,23,24]. However, there have been few studies of the relationships among dietary zinc intake, serum zinc concentration and multimorbidity in the Thai older population.

In the present study, we aimed to explore the associations among dietary zinc intake, serum zinc level, and multimorbidity in the geriatric population, and to evaluate serum zinc level and dietary zinc intake in older adults.

## 2. Materials and Methods

### 2.1. Study Design, Setting and Participants

The present study is a secondary analysis of data derived from 300 current and former employees of Electric Generating Authority of Thailand (EGAT) aged ≥ 60 years. All the participants volunteered to take part in a health survey on non-communicable disease risk factors and underwent clinical assessment with laboratory investigations. The study details and protocols of the EGAT study cohort have been previously described [25]. The current study is derived from the EGAT study survey conducted in 2013. Participants diagnosed with sepsis, autoimmune diseases, cancer, liver enzyme abnormalities or abnormal kidney function were excluded from the study. A liver enzyme abnormality was defined as a level of either aspartate aminotransferase (AST) or alanine aminotransferase (ALT) 5 × the upper limit of the normal range. Abnormal kidney function was defined using the upper limits of blood urea nitrogen (BUN) and serum creatinine concentrations. Participants were also excluded from this study if they did not complete a self-administered questionnaire or did not undergo physical examination, cognitive assessment or laboratory investigation.

### 2.2. Data Collection and Measurement Tools

During the visit, all participants completed a medical evaluation through comprehensive questionnaires, physical examinations and laboratory investigations. Twelve-hour-fasting serum samples were obtained from each participant. Individuals completed a self-reported questionnaire, and trained staff evaluated clinical performance and performed physical examinations. A comprehensive set of information was obtained from the EGAT database, which was compiled using data from self-reported questionnaires, interview-administered questionnaires, physical examinations, clinical assessments and laboratory investigations. In the study, the data from self-reported questionnaires included demographic characteristics, such as age, gender, marital status and income; lifestyle behaviors, such as current smoking status and alcohol drinking within the last 12 months; health information, such as chronic medical conditions and dental problems; and dietary intake and patterns. Interview-administered questionnaires were used to obtain information regarding cognitive function, mood and activities of daily living (ADL), and these were evaluated by trained personnel. Clinical measurements, including systolic blood pressure (SBP), diastolic blood pressure (DBP), waist circumference (WC), weight, height and body mass index (BMI), were also performed by trained medical staff.

#### 2.2.1. Structured Interview-Administered Questionnaire

A structured interview-administered questionnaire was used for the assessment of cognitive function, depression and ADL. Cognitive function was evaluated using the Montreal Cognitive Assessment (MoCA) test, on a scale of 0 to 30 [26], with lower scores indicating greater deterioration of cognitive function and a score of <21 referring to mild cognitive impairment [27]. With respect to psychological problems, depression was assessed using Thai Geriatric Depression Scale-15 (TGDS-15), which has a score range from 0 to 15, with a total score of >5 indicating depression [28]. Functional capacity and dependency status were evaluated using the Barthel Activity of Daily Living (BADL) index [29] and the Lawton Instrumental Activity of Daily Living (IADL) index [30]. The Barthel ADL index summarizes the performance abilities of individuals to use the toilet, feed themselves, perform self-grooming, dress, bathe, and their level of incontinence. Total scores range from 0 to 20, with lower scores reflecting higher levels of dependency. The Lawton IADL index assesses the necessity for assistance in the performance of eight activities, i.e., the use of a telephone, handling finances, food preparation, shopping, laundry, handling medications, housework and transportation, with summed scores ranging from 0 to 8. Higher scores indicate greater levels of ability.

#### 2.2.2. Clinical Measurements

Specially trained accessors measured SBP, DBP, BMI and WC. SBP and DBP were recorded as the means of two blood pressure assessment made using an automatic device placed on the left arm, while in a seated position, after a rest period of at least 5 min. Waist circumference was measured in a standing position midway between the lowest ribs and the superior border of the iliac crest [31]. The BMI was calculated by dividing the weight in kilograms by the square of the height in meters [32].

#### 2.2.3. Disease Definitions

Hypertension was defined as a positive history of hypertension, SBP ≥ 140 mmHg or DBP ≥ 90 mmHg [33]. Diabetes mellitus (DM) was described as a positive history of DM or a fasting glucose of ≥126 mg/dL [34]. Dyslipidemia was determined as a current history of dyslipidemia, a TC of ≥ 200 mg/dL, a TG of ≥150 mg/dL or an LDL-C of ≥ 130 mg/dL [35,36]. Participants were defined as alcohol drinkers or smokers if they consumed alcohol or smoked at the time of the study. In addition, participants recorded the presence of other diseases as “Yes” or the absence of these as “No” on the questionnaire.

#### 2.2.4. Dietary Assessment

Dietary assessment in the current study was conducted using a self-reported semi-quantitative food frequency questionnaire (SFFQ) and INMUCAL-Nutrients program of the Institute of Nutrition, Mahidol University [37]. The SFFQ recorded the average frequency of consumption of 40 food items on the basis of standard serving sizes during the preceding year.

#### 2.2.5. Biochemical Measurement

On the morning of the visit, fasting venous blood samples were collected with clotted blood, NaF and EDTA tubes for different target analyses. These serum samples were separated for subsequent analysis and stored frozen at −80 °C. White blood cell (WBC) count, hemoglobin (Hb), sodium, potassium, calcium, magnesium, phosphorus, uric acid, fasting blood sugar (FBS), triglyceride (TG), total cholesterol (TC), high-density lipoprotein-cholesterol (HDL-C), low-density lipoprotein-cholesterol (LDL-C), albumin, hemoglobin A1C (HbA1C) and Thyroid stimulating hormone (TSH) were measured through automated methods (Cobas-Mira, Roche, Milan, Italy). Homocysteine, vitamin B12 and folate concentrations were measured using an automated chemiluminescence method (Chiron Diagnostics, East Walpole, MA, USA). The protocol for serum zinc followed the modified method of Krachle et al. [38]. Serum zinc was measured using Agilent 7700x ICP-MS (Agilent Technologies, Santa Clara, CA, USA). In serum zinc analysis, 50 μL of zinc or blood reference control was supplemented with 200 μL of approximately 65% nitric acid (HNO3) and 100 μL of hydrogen peroxide (H_2_O_2_). Then, the mixture samples were placed in a water bath (60 °C) for 90 min. After cooling, the sample was supplemented with 2100 μL of ultrapure water for further analysis. The accuracy of ICP-MS was determined using Seronorm (Sero AS, Hvalstad, Norway) as a certified reference material. The accuracy and %CV of the LOQ was adjusted with ≤20%, and the target for the LOD was a half of the LOQ.

### 2.3. Statistical Analysis

The statistical analyses were performed with SPSS for Windows Software Package, version 25 (SPSS Inc., Chicago, IL, USA). The participants were classified into tertiles on the basis of their serum zinc levels (tertile 1 = 49.13−74.73 μg/dL or the lowest zinc levels; tertile 2 = 74.79−83.16 μg/dL or the medium zinc levels; tertile 3 = 83.18−150.56 μg/dL or the highest zinc levels). Data normality was evaluated with the Kolmogorov–Smirnov test. In terms of descriptive analyses, categorical data were reported as percentages and continuous data as means ± standard deviations (SDs) or medians ± interquartile ranges (IQRs). The correlation between the baseline–clinical characteristics and biochemical profiles, and serum zinc concentration was assessed using Pearson’s correlation analysis for normally distributed continuous data and Spearman’s rank correlation test for skewed continuous data. To identify univariate associations between serum zinc levels and clinical parameters, Pearson’s chi-square test for categorical variables, one-way analysis of variances (ANOVA) with the Tukey HSD post hoc test for normally distributed continuous data and Kruskal–Wallis test for non-normally distributed data were performed. Then, univariate and multivariate multinomial logistic regression analyses were further performed to determine significant associations between serum zinc and other clinical variables, using the highest tertile (T3) of zinc concentration as the reference category [39]. Potentially significant factors (*p*-value < 0.1 on univariate analysis) were included in the multivariate logistic regression analysis, which was performed using two models. Model 1 was adjusted for significant variables such as baseline age, sex, education, income, cognitive function and depression. Model 2 was further adjusted for the variables in Model 1, plus other potentially relevant clinical and biochemical parameters: BADL score, SBP, dietary zinc intake, and serum albumin, hemoglobin, homocysteine, triglyceride and calcium concentrations. The findings of the multivariate logistic regression analyses were reported as odd ratios (ORs) and 95% confidence intervals (CIs). *p*-values of <0.05 were regarded as indicating statistical significance.

### 2.4. Ethical Considerations

The present study was approved by Committee on Human Rights Related to Research Involving Human Subjects, Faculty of Medicine Ramathibodi Hospital, Mahidol University (protocol number: COA. MURA2021/862). All the participants were informed with respect to the objective, process, benefits and potential problems associated with the EGAT study. Then, written informed consent was obtained before their participation.

## 3. Results

### 3.1. Baseline and Clinical Characteristics, and Biochemical Parameters

Three hundred participants were enrolled in the study. The baseline characteristics were analyzed according to their tertile of serum zinc level, as shown in Table 1. The mean serum zinc concentration of all the participants was 80.5 (12.8) µg/dL, and their mean age was 63.0 (2.5) years, with a range of 60 to 71 years. One-fourth of the subjects were aged > 65 years. A minority of the study participants were female (*n* = 103; 34.3%) and had been in education for ≤12 years (*n* = 76; 25.3%). Approximately half of the total participants had an income of ≤200,000 THB per year. There were significant differences in the age, sex, educational level and income of the participants among the serum tertile groups (*p* < 0.05). Participants in the lowest tertile of serum zinc concentration were more likely to be >65 years of age, to have been educated for ≤12 years and to have an income of ≤ 200.000 THB per year than those in the highest tertile. In terms of lifestyle, 11% and 41.3% of the participants were smokers and alcohol drinkers, respectively, but there were no significant differences in the prevalence of these habits among the tertiles. The three most common chronic diseases were dyslipidemia (42.7%), hypertension (35.7%) and diabetes mellitus (14.7%). With regard to the psycho-cognitive status, the mean MoCA and TGDS scores of the participants were 25.3 (3.3) and 6.4 (1.5), respectively, but these did not differ among the tertile groups. However, the number of participants with cognitive impairment (MoCA score < 21) substantially increased as the serum zinc level decreased (T1−3 serum zinc of 19%, 13% and 7%, respectively; *p* = 0.041). In terms of dietary intake, the median daily zinc intake values of men and women were 4.5 mg/day (IQR 3.6−6.3) and 4.1 mg/day (IQR 3.1−5.2), respectively, and there was no relationship between dietary zinc intake and serum zinc level (*p* > 0.05). In addition, there were no differences in the mean WC, SBP, DBP or BMI among the three groups (*p* > 0.05).

The median TG concentration and the mean albumin, calcium, homocysteine and hemoglobin concentrations showed remarkable differences among the three groups (*p* < 0.05). However, only serum albumin, homocysteine and hemoglobin levels substantially increased with the increase in serum zinc levels, as presented in Table 2.

The present study revealed significant negative trends across the tertiles of serum zinc concentration with respect to age (r = −0.154, *p* = 0.008), BADL score (r = −0.115, *p* = 0.047) and SBP (r = −0.131, *p* = 0.023), as shown in Table 3. By contrast, we also found positive trends with regard to WC (r = 0.115, *p* = 0.048), serum TG (r = 0.153, *p* = 0.008), albumin (r = 0.403, *p* ≤ 0.001), calcium (r = 0.167, *p* = 0.004), uric acid (r = 0.131, *p* = 0.023), homocysteine (r = 0.248, *p* <0.001) and hemoglobin (r = 0.196, *p* = 0.001). In the subgroup analysis of participants with diabetes, hypertension or dyslipidemia, there were positive associations between serum zinc levels with serum albumin and homocysteine (*p* < 0.05). Although in participants with ischemic heart disease there were no associations of any parameter with serum zinc concentrations, in those who had experienced stroke, there were inverse associations between a number of parameters (serum cholesterol, LDL-C, calcium and vitamin B12) and serum zinc concentrations.

### 3.2. Factors Associated with the Serum Zinc Level

A multivariate logistic regression analysis was performed to identify independent factors that were associated with serum zinc concentrations, as shown in Table 4 and Table 5. Model 1 showed that the lowest tertile of serum zinc (49.13−74.73 μg/dL) was associated with being female (OR = 3.29; 95% CI: 1.72–6.32) and having been in education for ≤ 12 years (OR = 2.41; 95% CI: 1.09–5.29), compared with the highest tertile (83.18–150.56 μg/dL). After additional adjustment for clinical and biochemical parameters in Model 2, a significant association between depression (TGDS score > 5) and low serum zinc (49.13−74.73 μg/dL) was identified, presented as T1 vs. T3 (OR = 2.24; 95% CI: [1.06−4.77]). Additionally, increases in serum albumin (T1 vs. T3, OR = 0.01; 95% CI: 0.002−0.070) and triglyceride (T2 vs. T3, OR = 0.99; 95% CI: 0.98−0.99) was correlated with significant increases in serum zinc concentrations.

## 4. Discussion

In the present study, we revealed associations among dietary zinc intake, serum zinc level, and multiple comorbidities in people aged ≥ 60 years. In the geriatric population, the mean of serum zinc level was 80.5 (12.8) µg/dL, which corresponds to the findings of Barman et al. [40] but is higher than that found by Alqabbani et al. (70 (2.3) µg/dL) [41]. Approximately two-thirds of the population have been shown to be at risk of zinc deficiency (serum zinc concentration < 84 µg/dL) [39], which is one of the most common nutritional defects in older adults. In the aging population, zinc deficiency is most commonly caused by inadequate zinc intake, followed by intestinal malabsorption, chronic diseases and pharmacological interactions [18]. The majority of older people have a zinc intake < 50% of the recommended dietary allowance (RDA) for Thai adults aged > 60 years (8.6 mg/day for women, 10.9 mg/day for men) [42].

Although dietary zinc intake is not substantially correlated with serum zinc concentration [43], a minimum daily level of zinc intake is required to reach the RDA to maintain normal immune function and well-being [44]. A relationship between smoking or alcohol drinking and low serum zinc concentrations was not revealed in this study, in contrast to the results of recent studies [45,46]. It has previously been shown that tobacco smoke contains many oxidant compounds that can generate free radicals and increase oxidative stress [47]. Zinc acts as an antioxidant and is an essential co-factor in the oxidative defense mechanism [48]. Furthermore, cigarette smoking reduces appetite and dietary micronutrients intake [49]. Individuals with chronic alcoholism are at greater risk of inadequate dietary zinc intake, impaired absorption and greater urinary excretion of zinc, which have been reported to be causes of zinc deficiency in such individuals [50,51]. The most common comorbidity in older adults with low serum zinc concentrations was dyslipidemia, followed by hypertension and diabetes mellitus [21,52]. Nevertheless, there were no significant associations between these chronic diseases and low serum zinc levels, in contrast to the findings of preceding studies [53,54]. However, the relationship between metabolic syndrome and low serum zinc concentration was found in the preceding study [55]. In addition, the circulating concentrations of triglyceride, albumin, calcium, homocysteine and hemoglobin were positively correlated with serum zinc concentrations. This is consistent with the findings of Idei et al., in whose study low hemoglobin levels were found to be associated with low serum zinc concentrations [56]. Zinc plays an important role in red blood cell maturation. A zinc deficiency causes reductions in erythroid precursors in the bone marrow and in plasma erythropoietin [57,58]. Moreover, zinc deficiency may shorten RBC lifespan owing to an increase in reactive oxygen species (ROS) production [59,60]. A prior study showed a negative correlation between serum homocysteine and zinc concentrations [61]. Hyperhomocysteinemia induces oxidative stress through ROS production by increasing NADPH oxidase, resulting in low serum zinc concentrations [62].

Upon adjusting for the baseline characteristics of the participants in Model 1, we revealed that income and cognitive function were not associated with low serum zinc concentrations, which is similar to the findings of previous studies [63,64]. Model 1 identified the female sex and having been in education for ≤ 12 years as predictors of low serum zinc (49.13−74.73 µg/dL) [43,65,66,67]. Women are particularly prone to zinc deficiency following menopause [68], and the hormonal changes that occur during the perimenopausal period, including a depletion of estrogen, lead to increases in free radical generation and oxidative stress [69]. Estrogen serves as an antioxidant and maintains serum zinc concentration; therefore, low serum estrogen may be a cause of zinc deficiency. Previous studies have shown that the educational level influences smoking and alcohol consumption habits, physical activity and diet, all of which may contribute to zinc deficiency [70,71].

After additional adjustment for clinical and biochemical parameters in Model 2, a TGDS score > 5, which implied the presence of depression, was found to be a significant risk factor for low serum zinc concentrations (49.13−74.73 µg/dL), in line with the finding of Swardfager et al. [72]. The link between zinc status and depression can be explained through effects on serotoninergic pathways, given that zinc plays a major role in the regulation of serotonin receptors [73]. Additionally, ROS are involved in the pathophysiology of depression. Zinc can prevent ROS production and accumulation through numerous mechanisms, including the inhibition of nuclear factor kappa B and the activation of superoxide dismutase [74]. Furthermore, our study reported that high serum albumin concentrations were associated with high serum zinc levels, which is similar to the results of Hennigar et al. and Takahashi et al. [43,75]. This may be explained by serum zinc concentrations being affected by concentrations of zinc-binding proteins. Albumin is the primary protein responsible for zinc binding (80–85%), but alpha 2-macroglobulin (5−15%) and transferrin (<10%) [76] also contribute. In addition, it has been shown that low serum albumin is related to greater urinary excretion of zinc [76].

To the best of our knowledge, this is the first study to demonstrate the associations among serum zinc level, dietary zinc intake and multimorbidity among older adults in Thailand. This is similar to the study of Lu et al., which was the first study that reported on zinc nutrition status and on associated factors of zinc deficiency in the geriatric population in China [63]. The main strength of our study was the use of a comprehensive set of health data obtained from the EGAT database, which included basic characteristics, clinical and biochemical parameters, and dietary intake history. Furthermore, trained assessors conducted clinical assessments such as psychological conditions, cognitive status and activities of daily living. Nevertheless, a number of limitations of the study should be acknowledged. First, the current study included a relatively small number of participants, who were current and former employees of EGAT. Furthermore, older adults with many relevant diseases (sepsis, cancer or autoimmune diseases) were excluded from our study. Therefore, the findings may not be applicable to other geriatric populations. Second, causal relationships among serum zinc levels, dietary zinc intake and multiple comorbidities could not be assumed owing to the cross-sectional study design. Further longitudinal designs should be performed to address the limitation. Third, the use of a self-reported SFFQ for dietary assessment may be associated with recall bias. In addition, SFFQs are often limited in terms of the food items that are the main sources of dietary zinc. However, the use of this questionnaire is the most feasible and cost-effective method for gathering dietary data. Fourth, the serum concentrations of zinc and other metals are interrelated with each other; therefore, these complicated effects of mixture exposure should be assessed. In the present study, the concentrations of other trace minerals, such as copper, which may have affected serum zinc concentrations, were not taken into account. Finally, although adjusted multivariate multinomial logistic regression analyses were performed, the possibility of residual confounding could not be omitted.

Zinc supplementation could have advantages for geriatric population by promoting healthy aging and modifying susceptibility to chronic diseases through pro-inflammatory process and metallothionein (MT) [77]. The intervention for zinc deficiency prevention through the development of a comprehensive assessment tool including greater risk factors (serum albumin concentration and TGDs) should be implemented in clinical practice. In the future, the early diagnosis of chronic diseases could involve proteomic biomarkers related to inflammatory process or oxidative stress [78,79], which may be associated with zinc.

## 5. Conclusions

The independent factors associated with low serum zinc concentrations were found to be being female, having been in education for ≤ 12 years, having a TGDS score > 5 and having low serum albumin concentrations. Therefore, the early identification of these risk factors, as well as the management of comorbidities, including depression, and of low blood albumin concentrations, could help to prevent low serum zinc concentrations. In the current study, most of the older adults had inadequate daily zinc consumption; therefore, appropriate daily zinc intake based on the RDA should be encouraged.

## Figures and Tables

**Table 1:** Baseline and clinical characteristics of the participants classified by the tertile of serum zinc levels.

Characteristic	Total (n = 300) N (%)	Tertile of Serum Zinc Levels			
		T1 (*n* = 100) (49.13−74.73 µg/dL) N (%)	T2 (*n* = 100) (74.79−83.16 µg/dL) N (%)	T3 (*n* = 100) (83.18−150.56 µg/dL) N (%)	*p*-Value
Age, mean (SD)	63.0 (2.5)	63.7 (2.8)	62.7 (2.3)	62.7 (2.3)	0.019
≤65 years	243 (81)	72 (72.0)	85 (85.0)	86 (86.0)	0.001 *
>65 years	57 (19)	28 (28.0)	15 (15.0)	14 (14.0)	
Sex					
Male	197 (65.7)	54 (54.0)	64 (64.0)	79 (79.0)	0.001 *
Female	103 (34.3)	46 (46.0)	36 (36.0)	21 (21.0)	
Marital status					
Single	19 (6.3)	11 (11.0)	2 (2.0)	6 (6.0)	0.032
Married, windowed or separated	281 (93.7)	89 (89.0)	98 (98.0)	94 (94.0)	
Education					
≤12 years	76 (25.3)	37 (37.0)	25 (25.0)	14 (14.0)	0.001 *
>12 years	224 (74.7)	63 (63.0)	75 (75.0)	86 (86.0)	
Lifestyle factors					
Smoker	33 (11.0)	11 (11.0)	8 (8.0)	14 (14.0)	0.399
Alcohol drinker	124 (41.3)	35 (35.0)	42 (42.0)	47 (47.0)	0.223
Income					
≤200,000 THB per year	138 (46.0)	58 (58.0)	43 (43.0)	37 (37.0)	0.009 *
>200,000 THB per year	162 (54.0)	42 (42.0)	57 (57.0)	63 (63.0)	
Comorbidities					
Hypertension	107 (35.7)	31 (31.0)	39 (39.0)	37 (37.0)	0.470
Dyslipidemia	128 (42.7)	36 (36)	41 (41.0)	51 (51.0)	0.102
Diabetes mellitus	44 (14.7)	12 (12.0)	12 (12.0)	20 (20.0)	0.182
Ischemic heart disease	9 (3.0)	1 (1.0)	2 (2.0)	6 (6.0)	0.157
Ischemic stroke	3 (1.0)	1 (1.0)	1 (1.0)	1 (1.0)	1.000
Peripheral vascular disease	2 (0.7)	1 (1.0)	0 (0.0)	1 (1.0)	1.000
Dental problems	55 (18.3)	20 (20.0)	20 (20.0)	15 (15.0)	0.573
Waist circumference (cm), mean (SD)	86.1 (9.9)	85.1 (9.6)	85.7 (10.3)	87.4 (9.8)	0.255
Systolic blood pressure (mmHg), mean (SD)	142.8 (19.6)	142.9 (19.1)	145.7 (19.4)	139.7 (20.2)	0.097
Diastolic blood pressure (mmHg), mean (SD)	80.4 (10.9)	79.7 (11.6)	81.2 (10.9)	80.3 (10.2)	0.582
MoCA score, mean (SD)	25.3 (3.3)	24.9 (3.5)	25.3 (3.2)	25.5 (2.9)	0.463
MoCA score < 21	39 (13.0)	19 (19.0)	13 (13.0)	7 (7.0)	0.041
MoCA score ≥ 21	261 (87.0)	81 (81.0)	87 (87.0)	93 (93.0)	
TGDS, mean (SD)	6.4 (1.5)	6.5 (1.7)	6.5 (1.5)	6.1 (1.3)	0.096
TGDS ≤ 5	86 (28.7)	24 (24.0)	26 (26.0)	36 (36.0)	0.098
TGDS > 5	214 (71.3)	76 (76.0)	74 (74.0)	64 (64.0)	
BADL score, mean (SD)	19.9 (0.3)	20.0 (0.10)	19.9 (0.4)	19.9 (0.4)	0.033
IADL score, mean (SD)	7.9 (0.3)	7.9 (0.2)	7.9 (0.3)	7.9 (0.4)	0.661
BMI (kg/m2), mean (SD)	24.4 (3.7)	24.4 (3.9)	24.4 (3.8)	24.5 (3.3)	0.979
Dietary intake					
Dietary zinc intake (mg/day), median (IQR)	4.4 (3.4, 5.9)	4.5 (3.4, 5.9)	4.3 (3.3, 5.6)	4.6 (3.6, 6.1)	0.514

* *p* < 0.01. Data are presented as means (standard deviations), *n* (%) or medians (interquartile ranges). Abbreviations: SD, standard deviation; IQR, interquartile range; MoCA, Montreal Cognitive Assessment; TGDS, Thai Geriatric Depression Scale; BADL, basic activity of daily living; IADL, instrumental activity of daily living; BMI, body mass index; kg, kilogram; m, meter; cm, centimeter; mmHg, millimeter mercury; mg, milligram; µg, microgram; dL, deciliter.

**Table 2:** Biochemical parameters of the participants classified by the tertile of serum zinc levels.

Characteristic	Total (*n* = 300) N (%)	Tertile of Serum Zinc Levels			
		T1 (*n* = 100) (49.13−74.73 µg/dL) N (%)	T2 (*n* = 100) (74.79−83.16 µg/dL) N (%)	T3 (*n* = 100) (83.18−150.56 µg/dL) N (%)	*p*-Value
Cholesterol (mg/dL), mean (SD)	213.5 (44.6)	216.3 (46.3)	212.7 (41.7)	211.5 (46.1)	0.726
Triglyceride (mg/dL), median (IQR)	114.5 (85.8, 150.0)	107.0 (80.8, 143.5)	101.5 (81.8, 138.5.0)	128.5 (96.8, 170.5)	0.013
HDL-C (mg/dL), mean (SD)	58.5 (14.9)	57.8 (15.8)	60.4 (14.7)	57.5 (14.3)	0.328
LDL-C (mg/dL), mean (SD)	138.5 (40.7)	141.7 (39.7)	137.1 (39.1)	136.8 (43.5)	0.637
Glucose (mg/dL), mean (SD)	101.8 (22.2)	101.3 (20.8)	99.4 (18.4)	104.6 (26.5)	0.245
Total protein (g/dL), mean (SD)	7.4 (0.4)	7.4 (0.4)	7.5 (0.4)	7.5 (0.3)	0.111
Albumin (g/dL), mean (SD)	4.7 (0.2)	4.6 (0.2)	4.7 (0.2)	4.8 (0.2)	<0.001 *
Calcium (mg/dL), mean (SD)	9.6 (0.3)	9.6 (0.3)	9.7 (0.3)	9.7 (0.3)	0.041
Uric acid (mg/dL), mean (SD)	5.8 (1.4)	5.7 (1.3)	5.8 (1.5)	5.9 (1.3)	0.603
Phosphorus (mg/dL), mean (SD)	3.4 (0.5)	3.5 (0.5)	3.4 (0.5)	3.4 (0.5)	0.557
HbA1c (mg%), mean (SD)	5.8 (0.8)	5.8 (0.8)	5.7 (0.6)	5.9 (0.9)	0.182
TSH (µIU/mL), median (IQR)	1.8 (1.3, 2.4)	1.7 (1.3, 2.5)	1.8 (1.3, 2.4)	1.8 (1.3, 2.4)	0.370
Homocysteine (µmol/L), mean (SD)	15.7 (5.2)	14.2 (4.0)	15.8 (6.5)	16.9 (4.3)	0.001 *
Folic acid (ng/mL), median (IQR)	10.1 (7.7, 13.2)	10.4 (7.4, 12.5)	10.2 (8.1, 13.6)	9.9 (7.7, 13.2)	0.755
Vitamin B12 (pg/mL), median (IQR)	643.2 (494.0, 839.9)	651.6 (463.2, 829.7)	622.0 (520.2, 805.6)	633.1 (480.6, 846.5)	0.903
White blood cell count, (cells/mm^3^), mean (SD)	6.3 (1.6)	6.5 (1.5)	6.2 (1.8)	6.3 (1.6)	0.387
Hemoglobin, mean (SD)	14.0 (1.3)	13.6 (1.4)	14.0 (1.2)	14.4 (1.2)	<0.001 *
Platelet count (10^3^/mm^3^), mean (SD)	258.7 (66.6)	267.2 (62.4)	259.3 (78.6)	249.8 (56.1)	0.182

* *p* < 0.01. Data are presented as means (standard deviations) or medians (interquartile ranges). Abbreviations: SD, standard deviation; IQR, interquartile range; HDL-C, high-density lipoprotein-cholesterol; LDL-C, low-density lipoprotein-cholesterol; HbA1c, hemoglobinA1c; TSH, thyroid stimulating hormone; g, gram; mg, milligram; ug, microgram; ng, nanogram; pg, picogram; µIU, micro international unit; µmol, micromole; L, liter; mL, milliliter; dL, deciliter; mm, millimeter.

**Table 3:** Correlations between serum zinc levels and characteristics of the participants classified by comorbidity.

Characteristic	All (*n* = 300)		DM (*n* = 44)		HT (*n* = 107)		DLP (*n* = 128)		IHD (*n* = 9)		Stroke (*n* = 3)	
	r	*p*-Value	r	*p*-Value	r	*p*-Value	r	*p*-Value	r	*p*-Value	r	*p*-Value
**Baseline and Clinical Characteristics**												
Age	−0.154	0.008 *	−0.252	0.099	−0.186	0.055	−0.148	0.096	0.456	0.218	−0.991	0.085
BADL score	−0.115	0.047	−0.138	0.372	−0.120	0.217	−0.143	0.108	0.124	0.751	−0.134	0.915
IADL score	−0.054	0.365	0.081	0.613	0.056	0.584	0.049	0.599	0.002	0.976	0.008	0.884
BMI	0.017	0.767	0.037	0.810	0.064	0.514	0.065	0.469	−0.361	0.340	−0.954	0.195
MoCA score	0.051	0.380	0.269	0.078	0.136	0.162	0.115	0.195	0.069	0.860	0.134	0.915
TGDS score	−0.074	0.203	−0.135	0.383	−0.082	0.403	−0.082	0.356	−0.100	0.798	0.134	0.915
Waist circumference	0.115	0.048	0.087	0.574	0.164	0.091	0.131	0.141	−0.494	0.177	0.134	0.915
Systolic blood pressure	−0.131	0.023	−0.172	0.263	−0.087	0.370	−0.175	0.048	−0.253	0.511	−0.941	0.220
Diastolic blood pressure	−0.016	0.783	−0.186	0.228	−0.058	0.552	−0.041	0.645	−0.162	0.677	−0.841	0.365
**Dietary pattern**												
Dietary zinc intake	0.045	0.449	−0.221	0.159	−0.115	0.252	−0.081	0.378	0.167	0.668	−0.500	0.667
**Biochemical parameters**												
Cholesterol	−0.008	0.884	0.040	0.796	−0.065	0.508	0.038	0.667	−0.301	0.431	−1.000	0.015
Triglyceride	0.153	0.008 *	0.160	0.300	0.138	0.157	0.107	0.230	0.385	0.306	−0.500	0.667
HDL-C	−0.025	0.665	0.202	0.188	0.022	0.821	−0.029	0.742	−0.189	0.626	0.158	0.899
LDL-C	−0.002	0.976	0.059	0.706	−0.048	0.624	0.056	0.530	−0.326	0.392	−0.997	0.049
Glucose	0.052	0.366	0.061	0.693	0.103	0.290	0.069	0.440	−0.147	0.706	−0.974	0.145
Albumin	0.403	<0.001 *	0.523	<0.001 *	0.326	0.001 *	0.377	<0.001 *	−0.112	0.774	0.999	0.031
Calcium	0.167	0.004 *	0.034	0.826	0.130	0.181	0.103	0.249	−0.499	0.171	−0.998	0.036
Uric acid	0.131	0.023	0.145	0.348	−0.048	0.624	0.132	0.136	−0.300	0.432	0.951	0.200
Phosphorus	−0.032	0.578	0.087	0.573	0.052	0.592	0.074	0.409	−0.473	0.199	1.000	0.006 *
HbA1c	0.039	0.502	−0.127	0.413	0.003	0.973	0.071	0.425	0.057	0.884	−0.925	0.248
TSH	0.079	0.174	0.167	0.278	0.123	0.208	0.165	0.062	−0.167	0.668	1.000	0.010
Homocysteine	0.248	< 0.001 *	0.370	0.013	0.196	0.043	0.255	0.004 *	0.135	0.729	0.941	0.220
Folic acid	0.047	0.419	0.055	0.724	0.079	0.420	0.046	0.607	0.192	0.620	0.500	0.667
Vitamin B12	−0.003	0.962	−0.093	0.547	−0.096	0.326	−0.003	0.974	0.183	0.620	−1.000	0.010
Hemoglobin	0.196	0.001 *	0.115	0.456	0.165	0.089	0.176	0.047	−0.298	0.436	0.878	0.318

* *p* < 0.01. Data are presented as r (correlation coefficient) and *p*-value. Abbreviation: BADL, basic activity of daily living; IADL, instrumental activity of daily living; MoCA, Montreal Cognitive Assessment; TGDS, Thai Geriatric Depression Scale; BMI, body mass index; HDL-C, high-density lipoprotein-cholesterol; LDL-C, low-density lipoprotein-cholesterol; HbA1c, hemoglobinA1c; TSH, thyroid stimulating hormone; DM, diabetes mellitus; HT, hypertension; DLP, dyslipidemia; IHD, ischemic heart disease.

**Table 4:** Multivariate multinomial logistic regression analysis results (serum zinc levels in T1 vs. T3).

Characteristic	Model 1			Model 2		
	OR	95% CI	*p*-Value	OR	95% CI	*p*-Value
**T1 versus T3 (Reference)**						
Age > 65 years	1.724	0.792−3.750	0.170	1.789	0.721−4.441	0.210
Female	3.293	1.717−6.317	<0.001 *	1.340	0.529−3.397	0.537
Education ≤ 12 years	2.406	1.095−5.288	0.029	2.462	0.936−6.472	0.068
Income ≤ 200,000 THB per year	1.805	0.970−3.359	0.062	1.482	0.724−3.034	0.281
MoCA < 21	1.813	0.669−4.914	0.242	1.789	0.560−5.716	0.326
TGDS > 5	1.816	0.945−3.491	0.074	2.243	1.055−4.767	0.036
Dietary zinc intake (mg/day)				1.020	0.838−1.240	0.844
Systolic blood pressure (mmHg)				0.999	0.981−1.017	0.925
BADL score				6.523	0.808−52.638	0.078
Albumin (g/dL)				0.011	0.002−0.070	<0.001 *
Calcium (mg/dL)				1.426	0.403−5.046	0.582
Triglyceride (mg/dL)				0.996	0.989−1.002	0.202
Hemoglobin (g/dL)				0.822	0.591−1.142	0.242
Homocysteine (µmol/L)				0.975	0.904−1.052	0.514

* *p* < 0.01. Data are presented as odds ratios (95% confidence intervals). Model 1: Adjusted for age, sex, educational level, income, MoCA score and TGDS score. Model 2: Adjusted for age, sex, educational level, income, MoCA score, TGDS score, dietary zinc intake, systolic blood pressure, BADL score, and serum albumin, calcium, triglyceride, hemoglobin and homocysteine concentrations. Abbreviations: OR, odds ratio; CI, confidence interval; BADL, basic activity of daily living; MoCA, Montreal Cognitive Assessment; TGDS, Thai Geriatric Depression Scale; g, gram; mg, milligram; mmHg, millimeter mercury; dL, deciliter; L, liter; µmol, micromole.

**Table 5:** Multivariate multinomial logistic regression analysis results (serum zinc levels in T2 vs. T3).

Characteristic	Model 1			Model 2		
	OR	95% CI	*p*-Value	OR	95% CI	*p*-Value
**T2 versus T3 (Reference)**						
Age > 65 years	0.904	0.396−2.066	0.811	0.602	0.231−1.569	0.299
Female	2.130	1.121−4.046	0.021	1.459	0.601−3.539	0.404
Education ≤ 12 years	1.892	0.851−4.204	0.118	2.127	0.828−5.465	0.117
Income ≤ 200,000 THB per year	1.147	0.629−2.092	0.654	0.936	0.480−1.825	0.847
MoCA < 21	1.424	0.515−3.938	0.496	1.179	0.381−3.647	0.775
TGDS > 5	1.651	0.890−3.062	0.112	1.682	0.842−3.360	0.141
Dietary zinc intake (mg/day)				0.984	0.820−1.182	0.865
Systolic blood pressure (mmHg)				1.016	1.000−1.033	0.054
BADL score				0.599	0.251−1.432	0.249
Albumin (g/dL)				0.204	0.037−1.114	0.066
Calcium (mg/dL)				1.666	0.514−5.401	0.395
Triglyceride (mg/dL)				0.993	0.987−0.999	0.022
Hemoglobin (g/dL)				0.811	0.591−1.114	0.719
Homocysteine (µmol/L)				0.945	0.696−1.284	0.641

Data are presented as odds ratios (95% confidence intervals). Model 1: Adjusted for age, sex, educational level, income, MoCA score and TGDS score. Model 2: Adjusted for age, sex, educational level, income, MoCA score, TGDS score, dietary zinc intake, systolic blood pressure, BADL score, and the serum albumin, calcium, triglyceride, hemoglobin and homocysteine concentrations. Abbreviations: OR, odds ratio; CI, confidence interval; BADL, basic activity of daily living; MoCA, Montreal Cognitive Assessment; TGDS, Thai Geriatric Depression Scale; g, gram; mg, milligram; mmHg, millimeter mercury; dL, deciliter; L, liter; µmol, micromole.

## Data Availability

The data presented in the current study are not publicly available due to ethical restrictions. However, data are available from the corresponding author upon reasonable request.
